# Tailored Plasticization of Bio- and Fossil-Based Polymers Using a Versatile Bioplasticizer Derived from Phenylacetic Acid and Glycerol

**DOI:** 10.1021/acspolymersau.5c00149

**Published:** 2026-01-27

**Authors:** Laura Martellosio, Martina Ferri, Luca Lenzi, Arianna Tauro, Andrea Dorigato, Micaela Degli Esposti, Davide Morselli, Paola Fabbri

**Affiliations:** † Department of Civil, Chemical, Environmental and Materials Engineering, 9296 Università di Bologna, Via Terracini 28, Bologna 40131, Italy; ‡ 19034 National Interuniversity Consortium of Materials Science and Technology (INSTM), Via Giusti 9, Firenze 50121, Italy; § Department of Industrial Engineering, Università di Trento, Via Sommarive 9, Povo 38123, Italy

**Keywords:** phenylacetic acid, glycerol, plasticizer, biobased, biopolymers

## Abstract

For accelerating the shift from fossil-derived plastics toward biopolymers, there is an urgent need to develop efficient and versatile biobased plasticizers to improve biopolymer performance without compromising biodegradability and/or safety. This study explores the versatility of the emerging triphenylacetic glyceroate (TPAG) bioplasticizer by incorporating it into a range of biobased and conventional polymers. An increasing content of TPAG, from 5 to 20 parts per hundred of resin (phr), has been compounded with polyhydroxybutyrate (PHB), polyhydroxybutyrate-*co*-valerate (PHBV), polyvinyl chloride (PVC), and polybutylene succinate (PBS), which present complicated processability and/or limited mechanical properties as bare polymers. Differential scanning calorimetry reveals a clear reduction in glass-transition temperatures (*T*_g_) for PHB, PHBV, and PVC, with the most significant drop observed for PVC (Δ*T*_g_ = −25 °C at 20 phr TPAG), confirming the significant plasticizing efficiency of TPAG. A melting temperature decrease is also noted for PHB and PBS, with PHB exhibiting β-crystalline phase formation at high TPAG contents, which is attributed to enhanced chain mobility. Mechanical tests demonstrate that only 10 phr TPAG reduces Young’s modulus across all polymers, importantly enhancing their flexibility. Furthermore, 20 phr of TPAG increases the elongation at break of PVC and PHBV up to 349% and 22%, respectively. Volatility and migration studies demonstrate minimal plasticizer loss with values remaining well below safety limits. Moreover, TPAG addition also tailors both water contact angle and UV-blocking activity of the tested polymers, clearly indicating the versatility and multifunctionality of TPAG as a potentially suitable additive for consumer-facing applications.

## Graphical abstract

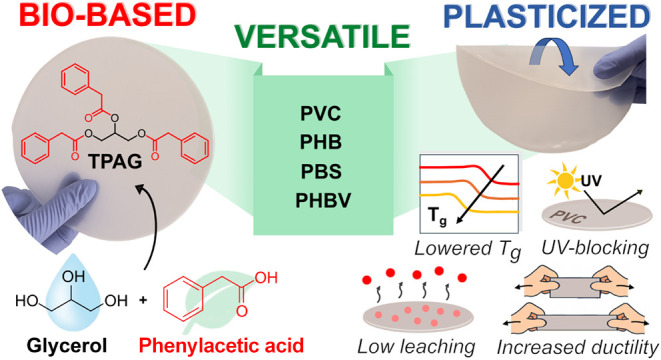

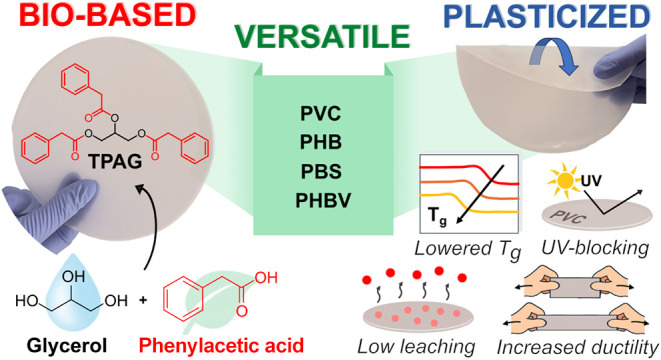

## Introduction

In the last decades, the environmental impact of fossil-based plastics and the need to reduce dependence on nonrenewable resources have been driving the shift toward bioplastics across various industrial sectors.^1^ Biopolymers such as polylactide (PLA), polyhydroxyalkanoates (PHAs), and polybutylene succinate (PBS) are gaining increasing attention in various applications, including food packaging,^2,3^ agricultural films,^4,5^ and biomedical devices,^6–8^ thanks to their renewability and biodegradability under specific conditions. However, their widespread use is still limited due to lower performance compared to their fossil-based counterparts, particularly in terms of mechanical, thermal, and rheological properties, which limit their processability and application versatility.

A simple and industrially appealing strategy to effectively overcome these disadvantages is the compounding of biopolymers with suitable additives. Representing more than 50% of the global additives market,^9^ plasticizers are essential additives widely used to tailor the mechanical, viscoelastic, and thermal properties of polymers. Among these, phthalates are the most commonly used due to their high plasticization efficiency, low cost, and versatility toward different polymers. However, increasing concerns have been raised in the last decades due to their environmental persistence, volatility, and potential toxicity,^10,11^ making them subject to stricter regulations and, in some cases, banned from certain applications (e.g., toys, food packaging, and biomedical devices).^12^ Indeed, combining biopolymers with fossil-based plasticizers leads to a significant lowering of the total renewable carbon content in the resulting compound, thus diverging from the sustainability goals outlined in current global policies.^13^ These considerations have raised interest in using natural-derived plasticizers with low toxicity and limited migration. This category includes epoxidized triglyceride oils from sources such as soybean,^14^ linseed,^15^ castor,^16^ and sunflower, as well as fatty acid esters and natural waxes.^17^ Moreover, increasing interest among material researchers and industries has been driven to the development of biobased plasticizers that can often offer better thermal stability and more consistent mechanical performance, thanks to their tailored molecular structure, which allows for stronger interactions with polymer matrices. Alternative plasticizers derived from biobased building blocks such as alkyl citrates, adipates, and epoxidized vegetable oils (EVOs) have already been proposed.^18^ However, many of these commercially available alternatives suffer from poor compatibility with a wide range of polymers, high migration rates, production costs,^18,19^ and still inadequate final mechanical properties compared to phthalates, hindering their practical use.^20^ For these reasons, there is an urgent need to develop versatile, low-migrating biobased plasticizers that can be effectively incorporated into different polymers while maintaining full sustainability and safety of the resulting material. Moreover, besides being nontoxic, highly miscible, low in migration, and efficient, ideal “green” plasticizers should also be cost-effective, an advantage that can be achieved by using waste or byproducts.

In recent years, glycerol triesters have emerged as one of the most promising families of bioplasticizers, which are derived from glycerol (GLY), a byproduct of the biodiesel supply chain, and organic acids, obtained from renewable or waste sources. These molecules can be synthesized by a sustainable synthesis route and offer remarkable plasticizing efficiency, which also positively affects the polymer processability in the molten state.^21^ Furthermore, some of these plasticizers have shown limited toxicity,^22^ fast biodegradation,^23^ and good compatibility with several polymers, making them attractive candidates for replacing conventional plasticizers in a broad range of applications.^22^

In this context, glycerol trilevulinate was synthesized through the solvent-free esterification of GLY and levulinic acid, which can be produced from lignocellulosic biomass.^22^ More recently, the synthesis of a novel biobased plasticizer, triphenylacetic glyceroate (TPAG), was also produced via solvent-free esterification of GLY with phenylacetic acid (PAA),^24^ which can be naturally found in plants as phytohormone with the function of a plant growth promoter.^25^ TPAG demonstrated not only promising plasticization effects but also it could add new functionalities to a polymer such as antioxidant, UV-blocking, and oxygen and water vapor barrier properties, which are fundamental for several applications.^24,26^ Based on these findings, the aim of this study is to investigate the versatility of TPAG by incorporating it at different concentrations into a broader range of polymers. Polyvinyl chloride (PVC) is a well-known and widely used fossil-based benchmark for evaluating plasticizer performance.^27^ Among biopolyesters nowadays considered valuable alternatives to conventional polymers, polyhydroxybutyrate (PHB), polyhydroxybutyrate-*co*-valerate (PHBV), and polybutylene succinate (PBS) are good candidates due to their limited processability and/or poor mechanical properties. Specifically, after TPAG incorporation into the polymer matrices through solvent casting, investigations on the films’ thermal, mechanical, morphological, and optical properties were performed. Moreover, migration in both polar and nonpolar solvents and volatility tests were conducted to evaluate TPAG’s leachability as a function of the polymer-additive compatibility.

## Experimental Section

### Materials

Methanol (99.8%), tetrahydrofuran (THF, HPLC grade), chloroform (CHCl_3_, HPLC grade), *n*-hexane (≥95%), ethyl acetate (EtOAc, 99.96%), water (HPLC grade), glycerol (GLY, ≥99%), phenylacetic acid (PAA, 98%), *p-*toluenesulfonic acid monohydrate (PTSA, 98.5%), sodium carbonate (Na_2_CO_3_, anhydrous, ≥99.0%), sodium chloride (NaCl, ≥99.5%), and sodium sulfate (Na_2_SO_4_, anhydrous, ≥99.0%) were purchased from Sigma-Aldrich and used as received, without further purification.

PHB (custom grade, *M*_n_: 106,200, *M*_w_: 425,900) and PHBV (custom grade, *M*_n_: 209,300, *M*_w_: 586,000, 20 mol % of 3HV) were purchased from Merck Group. The employed PHBV has a high amorphous content due to the incorporation of 3-hydroxyvalerate units, thus exhibiting negligible crystallinity, as evidenced in the DSC thermograms provided in the Supporting Information (Figure S1). PVC (industrial grade, *M*_n_: 64,900, *M*_w_: 150,300) was provided by Resilia Srl (Italy). PBS (industrial grade, *M*_n_: 53,200, *M*_w_: 146,300) was provided by NaturePlast (France).

Before the compounding and film preparation, all polymers were carefully purified by reprecipitation in order to remove all possible additives or impurities, which can alter the results. In detail, PVC was initially solubilized in THF (0.67 mg·mL^–1^), whereas PHB, PHBV, and PBS were solubilized in CHCl_3_ (0.67 mg·mL^–1^). All polymeric solutions were then vacuum-filtered on Celite and precipitated in a large excess of cold methanol, according to the procedure reported elsewhere.^8^ The plasticizer was synthesized optimizing the procedure reported in detail elsewhere.^24^ Briefly, the TPAG plasticizer was synthesized via esterification of PAA with GLY. GLY, PAA, and PTSA were mixed in a round-bottom flask with magnetic stirring and heated at 140 °C for 6 h. The reaction mixture was cooled, neutralized with saturated Na_2_CO_3_ solution, and extracted twice with EtOAc. The organic phase was then washed with Na_2_CO_3_ (aq) and brine (saturated aqueous solution), dried over anhydrous Na_2_SO_4_, and evaporated under reduced pressure, yielding a yellowish, viscous liquid in approximately 88% molar yield.

### Film Preparation

Neat and plasticized polymeric films were prepared by the solvent casting technique. Specifically, PHB, PHBV, and PBS were cast from CHCl_3_ solution (75 mg·mL^–1^), while PVC was cast from THF solution (75 mg·mL^–1^). TPAG plasticizer was added to the polymeric solution in 5, 10, and 20 parts by weight per hundred parts of resin (phr), then cast in a Petri dish (14 cm of diameter), and left to dry under the hood for 24 h at room temperature. As a result, PHB, PHBV, and PVC films of approximately 100 μm thickness were obtained and subsequently conditioned in a ventilated oven at 50 °C for 24 h to eliminate any residual solvent. For PBS-based samples, the obtained irregular films, after drying, were ground into a fine powder using a cryogenic mill and subsequently molded into uniform films using a hydraulic press with flat platens heated to 135 °C. Once pressure was applied for 5 min to ensure uniform film formation, the system was rapidly cooled by circulating cold water through the platens, resulting in PBS films approximately 100 μm in thickness. Neat films were used as references in all subsequent analyses. Film thickness was assessed by using a digital micrometer by taking measurements at five different positions on each sample and calculating the mean value and standard deviations, confirming minimal variability.

### Differential Scanning Calorimeter (DSC)

Thermal properties such as the melting temperature (*T*_m_), glass transition temperature (*T*_g_), and melting enthalpy (Δ*H*_m_) of neat and plasticized polymeric films were evaluated by DSC (Q10, TA Instruments) and fitted with a standard DSC cell, equipped with a Discovery Refrigerated Cooling System (RCS90, TA Instruments) and operated under a nitrogen atmosphere (purge flow: 20 mL·min^–1^). Approximately 3 mg of each sample were used. The first heating scan was performed from −60 to 190 °C at a rate of 20 °C·min^– 1^, followed by rapid cooling at the same rate. A second heating scan was then carried out at 10 °C·min^– 1^. For PBS samples, a modified protocol was used to obtain more accurate *T*_g_ detection. Specifically, samples were first melted and then rapidly quenched in liquid nitrogen to prevent full crystallization. Additionally, the first heating scan was performed at a higher rate of 30 °C·min^–1^ and with an initial temperature of −90 °C. DSC curves were processed with TA Universal Analysis 2000 software (TA Instruments). *T*_g_ values were extrapolated from the first heating scan, while *T*_m_ and melting enthalpy (Δ*H*_m_) were extrapolated from the second one.

Theoretical *T*_g_ values (*T*_g,FOX_) were calculated with the Fox Equation^28^ shown in eq 1:

1 $$1/T_{g,\mathrm{FOX}}=W_{1}/T_{g1}+W_{2}/T_{g2}$$

where *W*_1_, *T*_g1_, *W*_2_, and *T*_g2_ denote the weight fractions and *T*_g_ values of the plasticizer and polymer, respectively. The TPAG plasticizer presents its own *T*_g,DSC_ of −46 °C, as reported elsewhere.^24^

The *X*_c_ was calculated using eq 2:

2 $$X_{c}\left(\%\right)=\left[\Delta H_{m}\times 100\right]/\left[\Delta H_{m}^{0}\left(1-w_{p}\right)\right]$$

where *ΔH*_m_^0^ is the standard melting enthalpy of the 100% crystalline polymer and *w*_p_ is the weight fraction of the plasticizer in the sample. Selected *ΔH*_m_^0^ of PHB and PBS were 146 and 210 J·g^–1^, respectively.^29,30^

### X-ray Diffraction (XRD)

The phase composition and the crystalline structure of PHB and PBS films were investigated by X-ray diffraction performed on a PANalytical X’Pert PRO diffractometer equipped with a Cu-Kα ceramic X-ray tube (λ = 1.5418 Å) operating at 40 kV and 30 mA and with a fast X’Celerator detector. The characterizations were performed in air and at room temperature, in parallel-beam geometry and symmetric reflection mode (2θ range 5–55° 2θ, a step time of 564 s, and a step size of 0.1°/2θ). The measurement on each analyzed sample was repeated four times to reduce the signal noise. To ensure equal treatment across all samples and enable reliable comparisons, PHB and PBS films were preconditioned for 30 min in a hydraulic press with flat plates heated at 120 and 75 °C, respectively. The samples were then cooled to room temperature. Data were analyzed and processed by using PANalytical HighScore 5.1a software. In particular, the recorded XRD patterns were fitted by the pseudo-Voigt function in order to deconvolute overlapped diffraction peaks.

To better observe the variations of the peak area of a given crystallographic plane (*A*_ratio_[*hkl*]), the peak areas of a single phase (*A*_plast_[*hkl*]) of each compound were normalized by the corresponding peak area of the neat polymer (*A*_neat_[*hkl*]), according to eq 3

3 $$A_{\mathrm{ratio}}\left(\mathrm{hkl}\right)=\left(A_{plast}\left[hkl\right]\right)/\left(A_{neat}\left[hkl\right]\right)$$

### Tensile Tests

Mechanical properties of both neat and plasticized films were conducted using an INSTRON 5966 testing machine equipped with a 10 kN load cell and pneumatic grips. Rectangular-shaped specimens (10 × 70 mm^2^) were obtained from the prepared films. The crosshead speed was set to 4 mm·min^– 1^ during the testing procedure with a gauge length of 50 mm between the grips. The strain during tensile testing was determined from the crosshead displacement of the testing machine. Three specimens were tested for each sample to ensure the reliability and consistency of the results. Data analysis was performed using BlueHill Universal software.

### Scanning Electron Microscopy (SEM)

The morphology of the samples and the compatibility between the polymers and the plasticizer were evaluated by using field emission scanning electron microscopy (FE-SEM). The cryofractured cross-sections of the films were analyzed using a Nova NanoSEM 450 electron microscope (FEI Company, Bruker Corporation). All samples were previously coated with a thin layer of gold (approximately 10 nm) to ensure electrical conductivity. The obtained images were processed and analyzed using the open-source software FIJI—ImageJ.

### Optical Properties (UV–vis)

The optical properties of the films were analyzed using a VWR UV-6300PC spectrophotometer. Transmittance spectra of film stripes (10 × 30 mm^2^) were collected within the 200–800 nm spectral range. Transparency was evaluated based on the transmittance values measured at 600 nm. The UV-blocking activity was determined as follows:^24^

4 $$UV-X_{\mathrm{blocking}}=100-T_{UV‐X}$$

where *T*_UV‑X_ is the average transmittance value in the UV region, specifically from 200 to 400 nm. Each sample was measured in duplicate; the results were averaged, and standard deviations were calculated.

### Volatility and Migration Tests

The volatility of the plasticizer has been evaluated by storing film samples of approximately 50 mg (10 × 10 mm^2^) at 70 °C for 24 h. Volatility is expressed in terms of weight loss by eq 5, where *W*_1_ and *W*_2_ are the initial and final weights after the test, respectively.

5 $$Weight\;loss\left(\%\right)=\left[\left(W_{1}-W_{2}\right)\times 100\right]/W_{1}$$

The plasticizer migration resistance was evaluated by extraction tests in both deionized water and *n*-hexane according to international standard method ASTM D1239-14. In detail, 100 mg of each tested sample (approximately 15 × 15 mm^2^) was placed in a closed vessel containing 80 mL of extracting solvent for 24 h with gentle stirring at room temperature. Afterward, the samples were carefully dried for 24 h in an oven heated to 40 °C. The mass loss, due to plasticizer migration, was also calculated by eq 5, where *W*_1_ and *W*_2_ are the initial and final weights after the extraction test, respectively.

### Thermogravimetric Analysis (TGA)

The thermal stability of neat and 20 phr plasticized films was investigated by TGA (TGA5500, TA Instruments). Approximately 5 mg of each sample was placed in a platinum pan and analyzed through a temperature ramp of 10 °C·min^–1^ from 30 to 600 °C with a 60 mL min^–1^ nitrogen flow. Measurements were performed in duplicate. The weight loss of the samples as a function of temperature and the corresponding first derivative (DTGA) curves were recorded using TRIOS software (version 5.7).

### Water Contact Angle (WCA)

The surface wettability of the films was characterized by measuring static WCA. The measurements were made through the sessile drop method at room temperature using a contact angle goniometer (Krüss DSA 30, Nürnberg, Germany) equipped with a digital camera. For each sample, five 4 μL droplets of Milli-Q water were deposited at different locations on the surface, and side-view images of the drops were captured. Contact angles were automatically calculated by fitting the captured drop shape, measured after 30 s of equilibration after the drop deposition.

## Results and Discussion

As a proof-of-concept study, TPAG plasticizer has been incorporated into both a conventional fossil-based polymer (PVC) and a selection of biopolyesters (PHB, PHBV, and PBS) to assess its versatility and to enable a comparative evaluation of TPAG’s plasticizing efficiency and compatibility. TPAG was initially synthesized via a solvent-free reaction protocol previously optimized by our research group^24^ (Scheme 1A) and incorporated by the solvent casting method into the selected polymers at concentrations of 5, 10, and 20 phr, following the procedure shown in Scheme 1B.

**Scheme 1:**
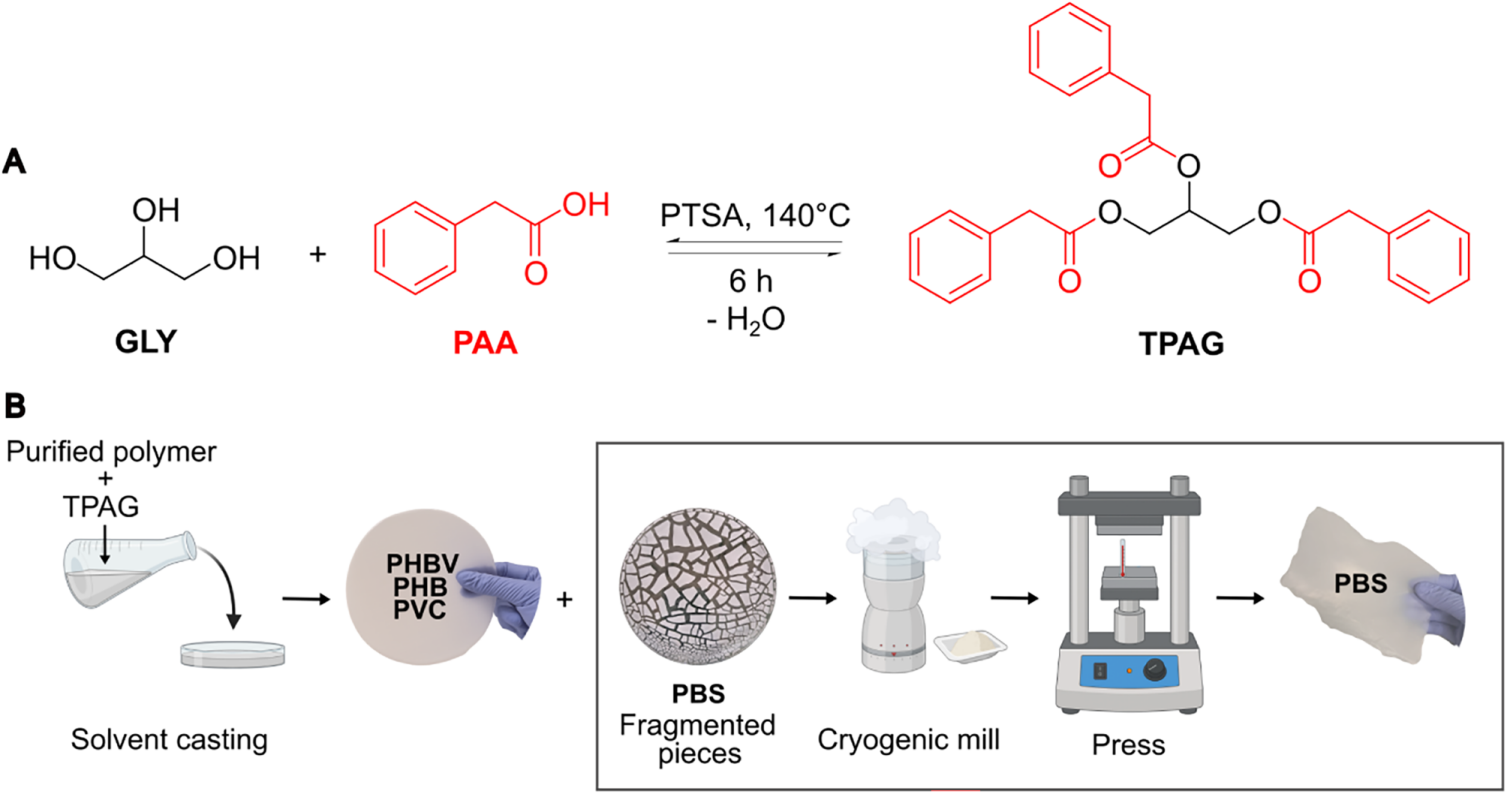
(A) Reaction Scheme of TPAG Synthesis; (B) Solvent Casting and Hot-Press Molding Process Use to Produce Neat and Plasticized Films

The modification of the thermal properties provides crucial insight into the efficiency and compatibility of plasticizers within polymers; therefore, DSC analyses were performed on neat and plasticized films. The obtained results were extrapolated from DSC thermograms (in Figures S2 and S3) and are summarized in Table S1 of the Supporting Information.

A key measure of a plasticizer’s effectiveness is its ability to lower the *T*_g_, which reflects a decrease in the intermolecular forces and internal friction between polymer chains.^31^ Therefore, Δ*T*_g,DSC_ values (Figure 1A) were calculated with respect to *T*_g,DSC_ of the neat polymer and plotted as a function of TPAG content. As the TPAG content increased, a progressive decrease in Δ*T*_g,DSC_ was observed across PHBV, PHB, and PVC formulations, demonstrating an efficient and versatile plasticizing effect. In particular, *T*_g,DSC_ of PHB decreased from 6 °C to −3 °C with an increase in the additive content from 0 to 20 phr, while for PHBV and PVC, it decreased from −2 °C to −9 °C and from 58 to 33 °C, respectively. The most pronounced reduction of Δ*T*_g,DSC_ was observed in PVC formulations, followed by a significant broadening of the transition range with the increase of TPAG content (DSC thermograms in Figure S2). This suggests that the plasticizer effectively lowered the energy needed to disrupt the interactions between polymer chains, even if the concentration of TPAG was relatively low, in good accordance with what has been previously reported using plasticizers with similar structures.^22,32^ However, TPAG had no significant effect on the Δ*T*_g,DSC_ of PBS formulations, which remained unchanged by increasing the TPAG content, suggesting a possible incompatibility between the polymer and the additive.

**Figure 1:**
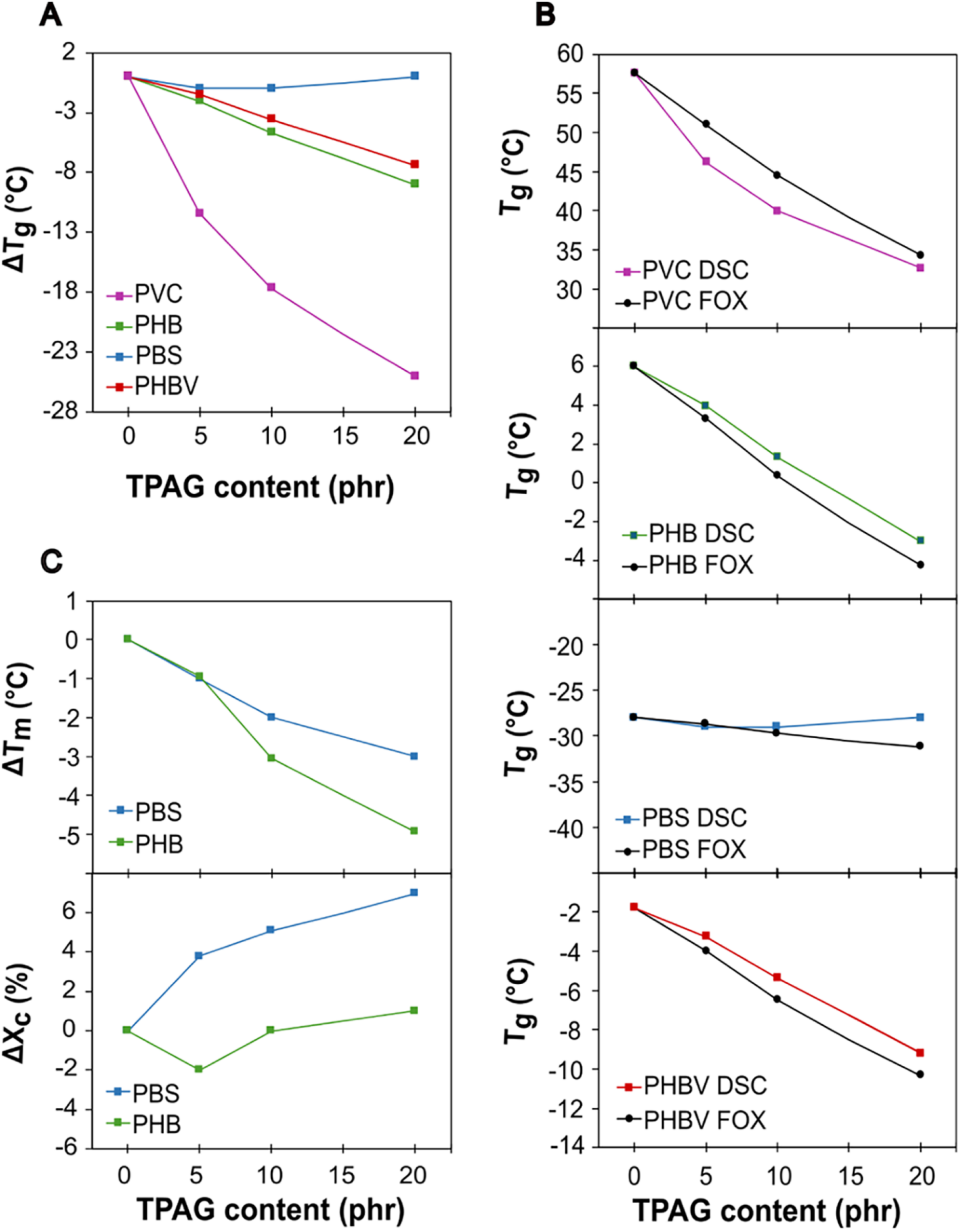
(A) Δ*T*_g_ as a function of plasticizer content for all prepared formulations. (B) *T*_g_ calculated using eq 1 (*T*_g,FOX_) and obtained from DSC investigations (*T*_g,DSC_), plotted as a function of the plasticizer content. (C) Δ*T*_m_ and Δ*X*_c_ of PBS and PHB formulations as a function of plasticizer content.

The extent of deviation between experimentally determined and theoretically predicted *T*_g_ values is an indicator of the plasticizer’s miscibility in the polymer matrix.^28^ Therefore, theoretical *T*_g,FOX_ was calculated following eq 1 and plotted, in comparison with experimental *T*_g,DSC_, as a function of TPAG content in Figure 1B. The values predicted by the Fox equation (eq 1) for all polymer formulations align well with the experimental *T*_g,DSC_ measurements, although the *T*_g,DSC_ of all plasticized PVC formulations was lower than its respective theoretical value. Moreover, a slight deviation between the experimental *T*_g,DSC_ and the theoretical *T*_g,FOX_ values was observed in PBS formulations containing more than 5 phr of TPAG, highlighting the negligible effect of the plasticizer in modifying the *T*_g_ of this polymer. This behavior can be attributed to the similarity between the *T*_g,DSC_ of neat PBS and TPAG, which likely hinders any substantial reduction in *T*_g_ upon blending. In addition to *T*_g_ reduction, which is representative of the thermal behavior of the amorphous region, the presence of a plasticizer can also affect the crystalline portion of a semicrystalline polymer.^32^ *ΔT*_m_ and *ΔX*_c_ of neat and plasticized formulation based on PHB and PBS were reported as a function of TPAG content in Figure 1C. PBS exhibited a slight decrease of *ΔT*_m_, by incorporating an increasing content of TPAG (115–112 °C from neat to 20 phr of TPAG formulation), whereas the crystallinity degree shows a modest increase (30% to 37% from neat to 20 phr of TPAG). On the other side, PHB showed a slightly more marked decrease of Δ*T*_m_, with *T*_m_ dropping from 172 to 167 °C for neat and 20 phr TPAG formulations, respectively. In addition, PHB exhibits the typical double melting peak, as shown in the DSC thermograms (Figure S3), with the lower peak at 147 °C becoming more prominent as the TPAG content increases. Considering that the polymeric films consist only of neat PHB and plasticizer, the double peak can only be attributed to the presence of two crystalline phases, as previously reported by Uzun et al.^33^ The DSC results possibly indicate a preferential growth of that respective crystalline phase by increasing the amount of TPAG. Furthermore, Δ*X*_c_ of PHB remains substantially unchanged with increasing TPAG content, with only a negligible decrease observed in the 5 phr formulation.

To better understand the influence of TPAG on the crystalline structure of semicrystalline polymers, XRD investigations were also performed on both neat and plasticized PHB and PBS. The results of the fitting and deconvolution processes obtained from the XRD patterns are reported in Figure S4 in the Supporting Information. The XRD results, presented in Figure 2, support the DSC findings, particularly in relation to the double melting peak observed in PHB, and helped clarify the effect of TPAG on polymer crystallinity (Figure S3). As mentioned above, PHB exhibits two crystalline forms, α and β, which coexist within the polymer. The α form has an orthorhombic unit cell and features greater lamellar thickness, while the β form, which has a noncentrosymmetric hexagonal crystal structure, has a smaller lamellar thickness and it typically arises with the physical stretching of the material.^34^ Compared to the α-phase, the β-phase is generally associated with increased flexibility, leading to an enhancement of the material ductility.^35^ Figure 2A shows XRD patterns associated with orthorhombic crystal planes that were identified using the PDF4+ database (00-049-2212) and assigned as [020], [110], [021], [111], [121], [040], and [002] at 13.5°, 17.0°, 20.1°, 21.5°, 25.6°, 27.2°, and 30.3°, respectively. On the other hand, the peak at 16.2° [011] and 22.6° are typically assigned to the β phase and were identified using the PDF4+ database (00-049-2213).^34^

**Figure 2:**
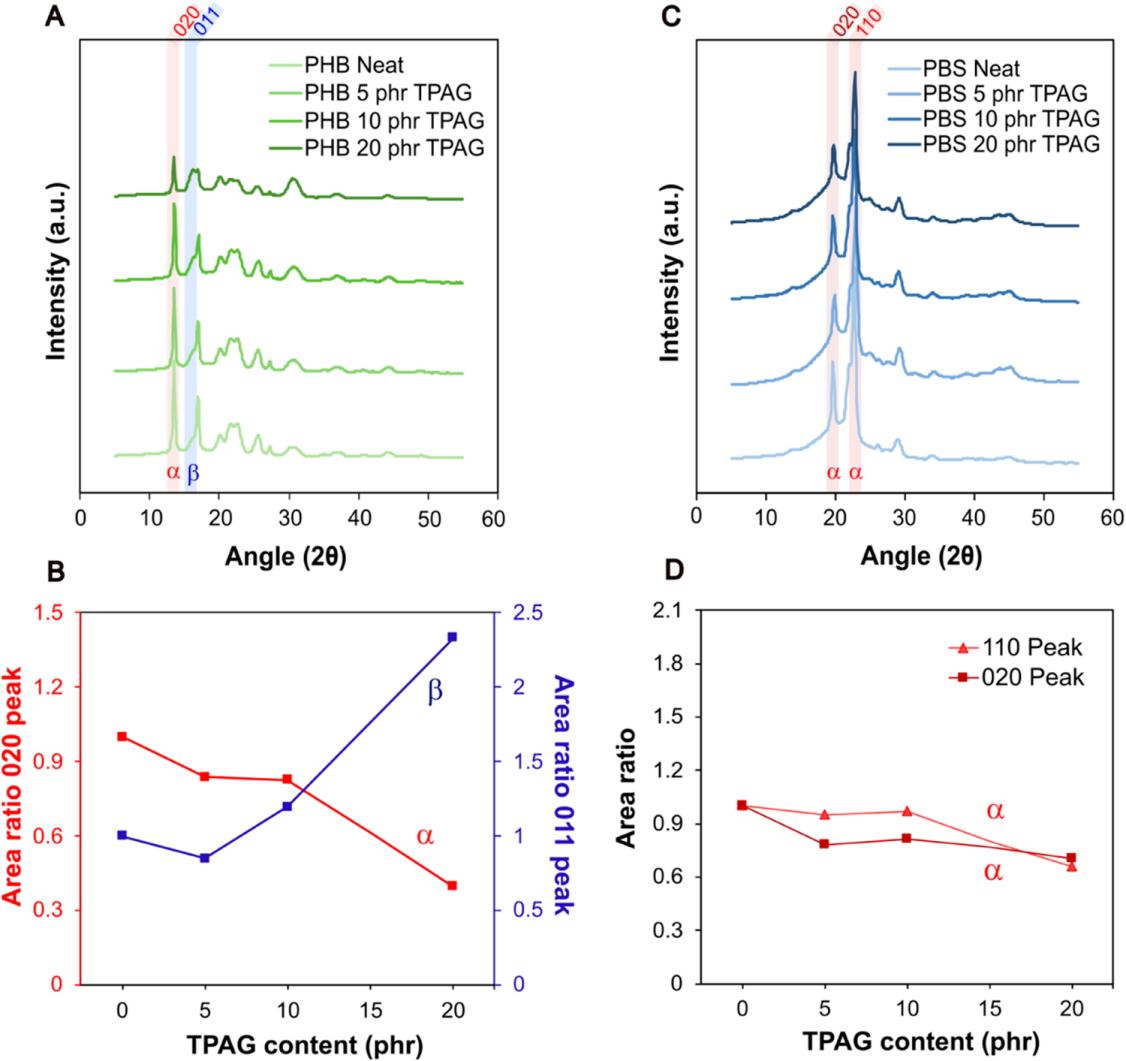
(A) XRD patterns of all of the PHB formulations. The most intense peak corresponding to the α-phase (020) is highlighted in red, while the most intense peak corresponding to the β-phase [011] is highlighted in blue. (B) Areas under the most intense peaks corresponding to the α-phase [020] in red and the β-phase [011] in blue, normalized to the peak area of neat PHB (by eq 3) and reported as a function of TPAG content. (C) XRD patterns of all PBS formulations. The two most intense peaks corresponding to the α-phase: [020] and [110] are highlighted in red. (D) Areas under the most intense peaks corresponding to the α-phase: [020] and [110] normalized (by eq 3) to the peak area of neat PBS and reported as a function of TPAG content.

To investigate the relative changes in crystalline phases, the areas under the most intense peaks corresponding to the α-phase [020] and β-phase [011], normalized to the peak area of the relative peak of neat PHB (by eq 3), were plotted as a function of TPAG content (Figure 2B). The normalized single peak area ratios indicate a gradual decrease in the α-phase [020] and a concurrent increase in the β-phase [011] with rising TPAG content, suggesting a qualitative shift toward β-phase formation.

This trend may be attributed to the plasticizing effect of TPAG, which likely increases the free volume among the PHB chains. According to the free volume theory,^36^ the resulting enhancement in chain mobility could facilitate the structural rearrangements needed for the development of the β-crystalline phase.

Also, PBS typically exhibits a single crystalline phase under standard processing conditions, the α-phase with a monoclinic crystal structure, while the β-phase is generally induced through physical stretching of the material similarly to what was previously discussed for PHB.^37,38^ In the XRD patterns of PBS films (Figure 2C), the most intense diffraction peaks appear at 19.6°, 22.0°, and 22.7°, corresponding to the [020], [021], and [110] crystallographic planes, respectively.^39^ Comparison with reference data in the literature confirms that these peaks are characteristic of the α-crystalline phase of PBS, indicating the exclusive presence of the α-crystalline phase in all PBS samples, regardless of plasticizer content.^38^ The single peak area ratio of [020] and [010] phases (Figure 2D) indicates no modification of the crystalline structure with the increase of TPAG content, which is in line with what was previously described for PBS thermal properties. It is reasonable to assume that the higher the density of polar groups in the structure of the polymer, the higher the number of interactions with carboxyl groups of TPAG.^22^ In the case of PBS, its relatively low polarity, resulting from its aliphatic backbone and limited presence of polar functional groups, reduces its affinity for TPAG, thereby hindering the plasticizer’s effective incorporation and potentially explaining its negligible impact on the overall behavior of the PBS-based system and the slight antiplasticizing effect observed at higher TPAG loadings.^40^

As known, the incorporation of the synthesized plasticizer influenced not only the thermal behavior of the compounds but also their mechanical properties. Basically, the intercalation of the plasticizer molecules among polymeric chains results in the disruption of the intermolecular forces, which increases chain mobility and thus material ductility.^41^ This effect macroscopically enhances the overall flexibility of the material, making it more adaptable to mechanical deformations. To assess these effects, tensile tests were conducted on all plasticized samples, with neat formulations being used as a reference. The stiffness of each formulation was evaluated through Young’s modulus (*E*), while elongation at break (ε_break_) was measured to determine improvements in flexibility (Figure 3A). Representative stress–strain curves of the neat and plasticized formulations are presented in Figure S5 and Table S2 in the Supporting Information.

**Figure 3:**
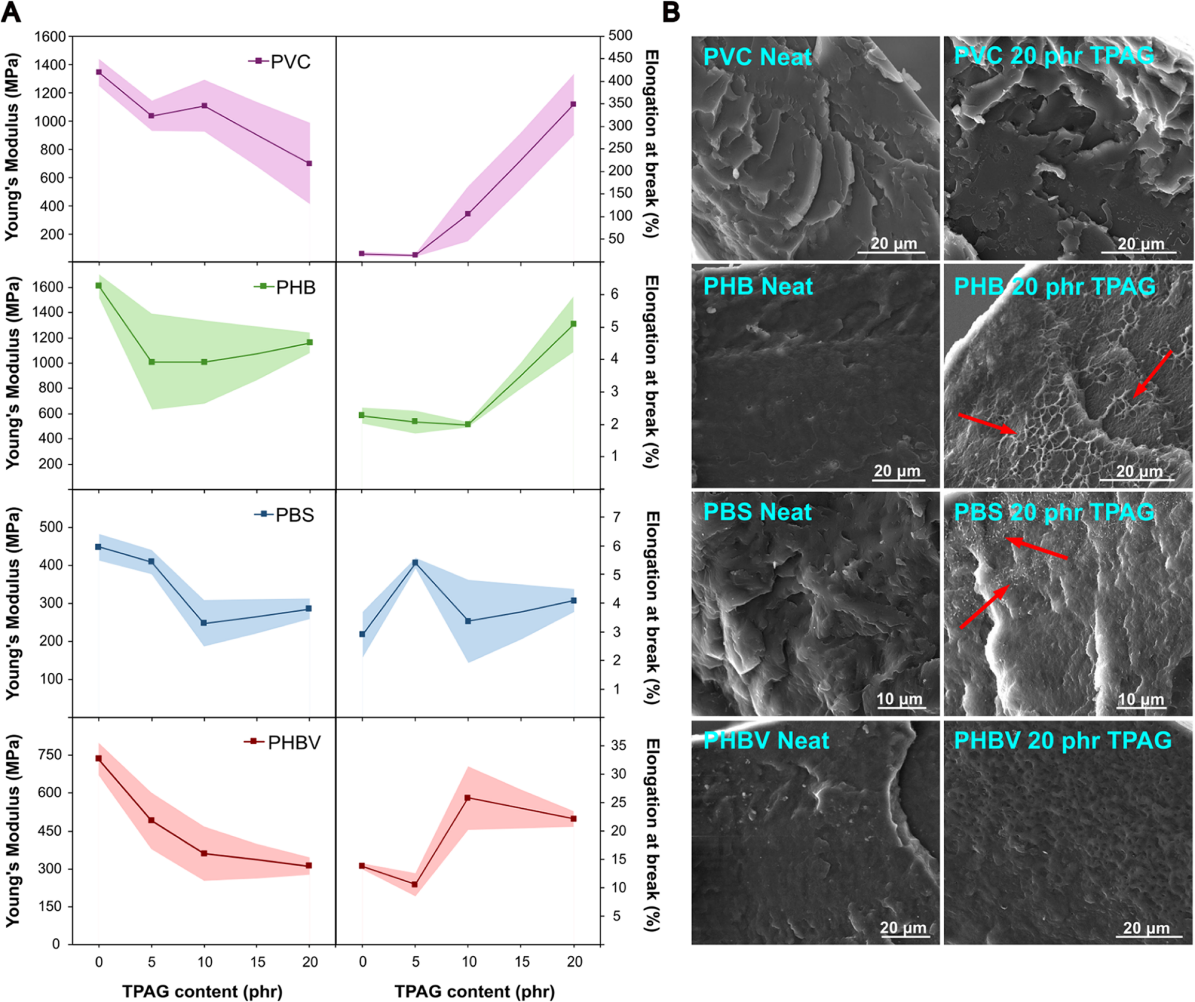
(A) Young’s modulus (E) and elongation at break (ε_break_) for each prepared polymeric compound as a function of TPAG content. The shaded area represents the standard deviation of the measurements. (B) Cross-sectional FE-SEM micrographs (secondary electrons) of neat and 20 phr formulation PVC, PHB, PBS, and PHBV films. Red arrows denote the evidence of ductile behavior/fracture in plasticized samples.

In detail, the *E* of PVC slightly decreased from 1346 MPa (neat polymer) to 1035 and 1107 MPa in the 5 and 10 phr formulations, respectively. A significant reduction in *E* was observed when 20 phr of TPAG was incorporated in PVC, which dropped to 697 MPa (Figure 3A). This reduction in stiffness was also observed as an important increase in the elongation at the break. While the neat polymer exhibits an ε_break_ of only 17%, this value impressively raised to 349% in the 20 phr formulation (Figure 3A), outperforming the mechanical properties obtained with some commercial fossil-based plasticizers, such as di­(2-ethylhexyl) phthalate (DEHP).^42^ The *E* of PHB decreased from 1610 MPa of the neat polymer to 1160 MPa in the 20 phr formulation (Figure 3A), likely due to structural changes observed through XRD analysis (Figure 2B). Meanwhile, ε_break_ increased from 2.5% (neat PHB) to 6% in the 20 phr formulation (Figure 3A), indicating an important improvement in flexibility, especially considering the well-known PHB brittleness and the difficulties to contrast it. Concerning PBS, the *E* values slightly decreased from 447 MPa in the neat polymer to 248 and 285 MPa in the 10 and 20 phr formulations (Figure 3A), respectively. However, ε_break_ remained almost unchanged across all formulations, with a modest increase observed only in the 5 phr formulation (5%), highlighting the plasticizer’s limited capacity to further improve ductility beyond that concentration. For PHBV, *E* consistently decreased with the TPAG content, dropping from 736 MPa for the neat polymer to 310 MPa for the 20 phr formulation (Figure 3A). Meanwhile, a noticeable increase in ε_break_ occurred in the formulations containing more than 10 phr of TPAG, rising from 14% in the neat polymer to 26% and 22% in the 10 and 20 phr formulations (Figure 3A), respectively.

SEM investigations were employed to investigate the morphology of the materials, with particular attention paid to plasticizer miscibility and potential surface migration, in order to elucidate the mechanisms underlying the observed mechanical behavior. SEM imaging was performed on both neat polymers and formulations with the highest plasticizer content (20 phr), since they are the samples that have the highest tendency to leach out. As shown by Figure 3B, all the formulations exhibited good phase homogeneity, as no additive droplets were observed on the matrices, indicating the absence of phase separation. In detail, PVC and PHBV plasticized samples exhibited no morphological differences between the neat and plasticized formulation. This lack of visible differences is expected for systems in which the plasticizer is highly compatible with the host polymer. When the plasticizer is well dispersed and interacts only within the amorphous region, it increases chain mobility and lowers intermolecular interactions without generating microstructural features detectable by SEM. As a result, fracture surfaces of neat and plasticized PVC or PHBV may appear similar, even though their mechanical behavior is significantly altered by plasticization. Although the cross sections were obtained under cryogenic conditions, a ductile fracture behavior is observed with higher TPAG content in PHB, which is evident from the greater number and length of ductile regions, as highlighted by the red arrows in Figure 3B. Such behavior was expected, as the incorporation of TPAG lowered the *T*_g_ and the stiffness of PHB, enhancing its ductility, thus confirming its good plasticizing effect on this particularly brittle polymer. For PBS, ductile features were also observed but were largely restricted to the surface and appeared less pronounced overall, likely due to differences in film processing. These surface–bulk morphological differences may account for the limited effect of TPAG on this polymer, as displayed in the mechanical tests (Figure 3A).

Since transparency is an important factor in various material applications, such as food packaging, biomedical devices, and coatings, it is essential to evaluate the optical properties of neat and plasticized films to assess their suitability for the intended application. Figure 4A presents the final appearance of the films, highlighting the differences between the neat films and those incorporating 20 phr of plasticizer. The transparency of PHB, PHBV, and PBS remains largely unaffected by the incorporation of TPAG. In contrast, PVC exhibits a decrease in transparency, dropping from 98% to 63% (evaluated as transmittance at 600 nm), which is also visible from the UV–vis spectra in Figure 4B. To compare the effect of TPAG on the transparency of each studied polymer, the transmittance at 600 nm was extrapolated and reported in Figure 4C. Except for neat PVC, all the formulations analyzed fall within the category of translucent materials, exhibiting a transparency lower than 80% (Figure 4C).^43^

**Figure 4:**
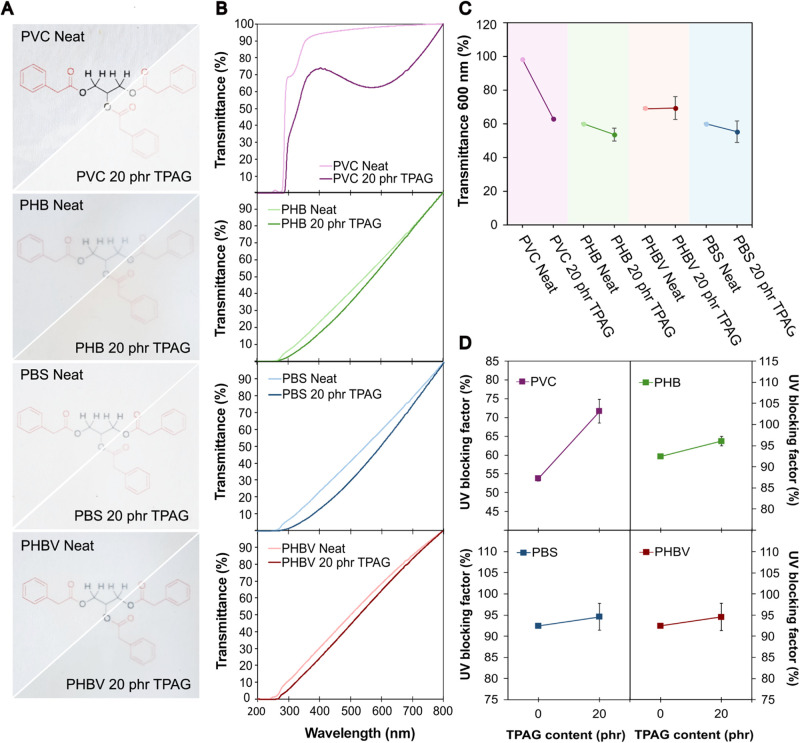
(A) Photographs showing the appearance of films of neat and 20 phr TPAG formulation of PVC, PHB, PBS, and PHBV. (B) UV–visible spectra, (C) transmittance values at 600 nm and (D) UV-blocking factor of neat polymers and related 20 phr TPAG formulations.

Due to its π-conjugated aromatic system that can absorb UV irradiation, TPAG had previously demonstrated its potential UV-blocking effect when incorporated in high concentration in polyesters.^24^ Therefore, the UV-blocking activity was calculated using eq 4 for neat and 20 phr TPAG films (Figure 4D). Due to their intrinsic molecular structure, characterized by the presence of carboxylic groups in the polymer backbone, PHB, PHBV, and PBS exhibited very high UV-blocking factors already as bare material, exceeding 90%, which only slightly increases by the addition of the plasticizer (Figure 4D). In contrast, PVC, which is a polymer that typically suffers from UV radiation, showed an increase in UV blocking when TPAG is added, rising from 54% to 72% with the addition of 20 phr of TPAG (Figure 4D).

Plasticizer volatility and migration are critical aspects that influence the long-term performance, safety, and environmental impact of plasticized materials. Many conventional and biobased plasticizers suffer from significant loss during use, which can lead to a drop of the desired properties and potential health or ecological risks.^44^ To assess the stability and retention of TPAG within different polymers, volatility and migration tests were performed. Thermal stability was specifically evaluated by measuring weight loss due to volatilization (Figure 5A), which is influenced by molecular weight, chemical structure, and compatibility of the additive with the polymer.^45^ This analysis is essential to determine the suitability of TPAG for applications involving thermal stress and prolonged air exposure. The weight loss (calculated by eq 5) due to volatility of TPAG was negligible (Figure 5A). For all the plasticized formulations of PVC, PHB, and PHBV, TPAG exhibited low volatility, which never exceeded 2.2 wt % and reached its minimum at the highest TPAG concentrations. This behavior can be attributed to the fact that, at high plasticizer concentrations, not only polymer–plasticizer interactions increase, but interactions between plasticizer molecules also become more significant, creating a more viscous and denser environment.^23^ Such conditions may hinder molecular mobility and reduce the tendency of plasticizer molecules to migrate toward the polymer surface, thus limiting their volatility. In contrast, PBS displayed consistently higher volatility values, around 5 and 6%, regardless of the plasticizer content. SEM analysis (Figure 3B) revealed that plasticization in PBS occurred primarily at the surface, with no evidence of bulk incorporation, making the plasticizer more exposed to heat and thus more prone to be released.

**Figure 5:**
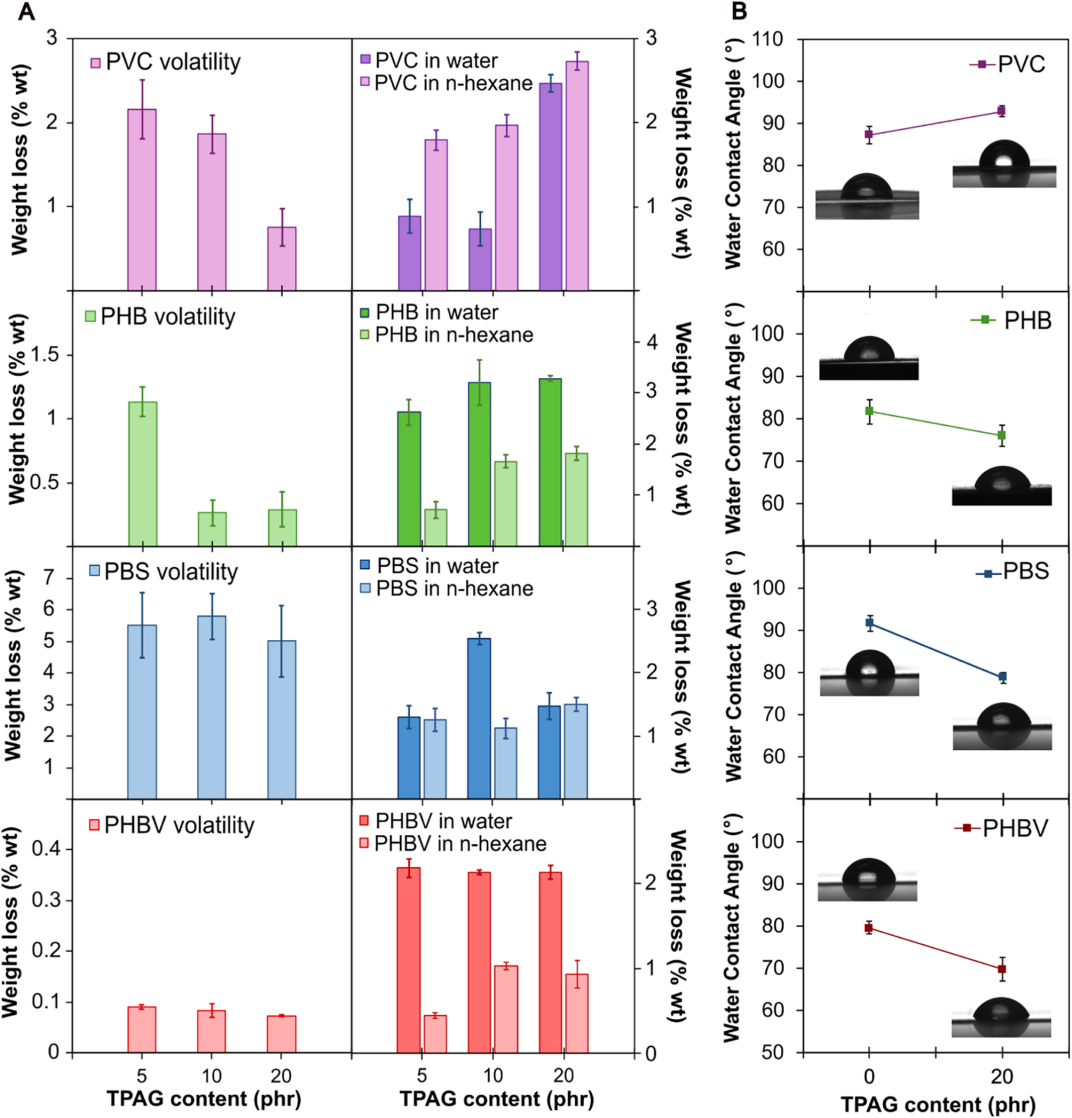
(A) Weight loss of plasticized films evaluated by volatility tests and by an extraction test in water and *n*-hexane. (B) Water contact angle (WCA) of neat and 20 phr TPAG formulations as a function of the TPAG content. Representative photographs of water droplets on the film surface were reported close to the associated point on the graph.

To further investigate the thermal stability of the neat and plasticized films, TGAs were conducted on both neat and 20 phr TPAG formulations of all four tested polymers (thermograms in Figure S6 of the Supporting Information). Regardless of the polymer, the additive incorporation did not affect the degradation temperature of the materials, confirming that TPAG maintains the thermal integrity of the polymer while ensuring low volatility.

In both solvents, the neat polymers exhibited minimal leaching (calculated by eq 5), consistently below 1 wt %, which can be attributed to the release of low molecular weight fractions inherently present in the polymer matrices. Overall, all the plasticized formulations showed low migration levels in both selected solvents (not exceeding 3.3 wt %), indicating good retention of TPAG within the polymeric networks, despite the testing conditions being selected to force the additive migration.

According to the European Union Regulation 2023/1442,^46^ the tolerable daily intake (TDI) of phthalates, such as DEHP, is set at 50 μg·kg^–1^ of body weight. This value represents the maximum amount that can be safely assimilated daily throughout a lifetime without posing health risks, corresponding to a limit of 3.5 mg/day for an adult weighing 70 kg. To reach this level of exposure from the tested materials, more than 90% of TPAG would need to migrate from the polymer within 24 h of immersion, which greatly exceeds the weight losses observed.

Consistent with these findings, the safety of TPAG has been previously investigated in the context of PLA-based food packaging.^24^ In that study, migration tests were conducted in accordance with the EU Technical Guidelines for compliance testing under the framework of Plastic FCM Regulation (EU) No. 10/2011, using Tenax^Ⓡ^ as a simulant for dry food. The results demonstrated that, except for the formulation containing 20 phr of TPAG, the overall migration values of all samples were below 10 mg·dm^–2^, corresponding to the maximum limit established by current European legislation for food-contact materials. Nevertheless, a comprehensive toxicological assessment, including dedicated in vitro and in vivo studies, would be required to fully evaluate the safety profile of the TPAG plasticizer.

All polyester-based formulations (PHB, PHBV, PBS) exhibited higher plasticizer migration in water compared to *n*-hexane, exhibiting a weight loss that ranges from 0.02 to 3.30 wt % (Figure 5A). This behavior can be attributed to the intrinsic polarity of the plasticizer, which bears three ester groups in its molecular structure that enhance its affinity for aqueous environments.

In order to further understand the interactions between the plasticizer and the different polymers, the hydrophilicity and hydrophobicity of the polymeric films were assessed by measuring the water contact angles of both neat and 20 phr TPAG-plasticized formulations, as reported in Figure 5B. PVC formulations exhibited a slight increase in the WCA from 87° to 92° (Figure 5B). This result suggests that TPAG remains more uniformly distributed within the bulk or interacts with the polymer in a way that promotes the orientation of its apolar aromatic chains toward the surface, thereby enhancing the surface’s hydrophobic character. An alternative explanation for this phenomenon is that the plasticizer can shield the polar C–Cl groups along the PVC backbone either physically or through electrostatic effects, thereby reducing their potential interaction with water molecules.^47^ The reduced surface affinity for water molecules may also explain the higher extraction of TPAG from PVC into hexane compared to that from water. In contrast, a slight decrease in WCA was observed for all polyester-based systems, indicating an increase in surface hydrophilicity. This effect is likely due to the formation of polar interactions between the ester groups of TPAG and those of the polyesters, which promote good compatibility and the surface orientation of TPAG’s polar ester functionalities, thereby enhancing the surface affinity for water. This effect is particularly relevant for polymers such as PHB and PHBV, which are widely proposed as biomaterials for tissue regeneration^48^ but whose poor hydrophilicity limits their applicability.

## Conclusions

This study demonstrated the effectiveness and versatility of the biobased TPAG plasticizer in modifying the thermal, mechanical, and physico-chemical properties of both fossil- and bio-based polymers, such as PVC, PHB, PHBV, and PBS. TPAG incorporation led to a progressive reduction in *T*_g_ for PHB, PHBV, and notably for PVC, confirming its efficient plasticizing effect through enhanced chains mobility by reducing intermolecular forces. Concerning PBS, a slight *T*_g_ reduction was observed only at the 5 phr formulation, which appeared to be the most effective loading, as higher concentrations did not yield further changes. Additionally, decreases in *T*_m_ were observed for semicrystalline PHB and PBS. Taking into account the typical narrow processability window of PHB in the molten state, even the relatively small lowering of *T*_m_ obtained offers a potential improvement in material handling and processing. Moreover, PHB exhibited the formation of a β-crystalline phase at high TPAG content, as revealed by DSC and XRD analyses, which was attributed to increased chain mobility. Mechanical testing showed that the addition of only 10 phr TPAG could substantially reduce Young’s modulus across all polymer systems, resulting in enhanced flexibility at room temperature. With 20 phr plasticizer incorporation, TPAG significantly enhanced the elongation at break of PVC, reaching 349%, indicating excellent plasticization efficiency. The ultimate elongation improvements in PHB and PHBV were less pronounced (6 and 25%, respectively), but they still represent significant gains in flexibility and are important for enhancing the overall processability and typical brittleness of these materials. In the case of PBS, a small increase in elongation at break (up to 5%) was observed only at the 5 phr formulation, further supporting the trends identified through thermal analyses. Moreover, SEM investigations confirmed good homogeneity between the synthesized plasticizer and the selected polymers and the absence of phase separation across all formulations, supporting the effective dispersion and compatibility of TPAG with the polymers. Volatility and migration studies revealed low plasticizer losses in all the tested polymers, even at elevated TPAG loadings and forced leaching conditions, demonstrating a remarkable compounds’ stability. Specifically, all losses due to migration remained well below critical exposure thresholds, suggesting the materials’ potential safety for consumer-related applications.

Overall, TPAG proved to be a promising and versatile biobased plasticizer, capable of enhancing flexibility and thermal processability across various polymer systems. Additionally, its ability to modify surface wettability and impart UV-blocking properties highlights its potential not only as an alternative to commercial plasticizers, but also as a multifunctional additive.

## Supplementary Material



## Notes

Figure S1: Full DSC thermograms of PHBV/TPAG formulations; Figure S2: DSC thermograms, specifically showing glass transition temperatures of neat polymers and TPAG formulations; Figure S3: DSC thermograms, specifically showing melting temperatures of neat PHB and PBS and related TPAG formulations; Figure S4: XRD patterns of PHB and PBS formulations; Figure S5: Stress–strain curves of neat and plasticized samples; Figure S6: TGA thermograms of the neat polymers and formulations with 20 phr of TPAG; Table S1: Summary of the thermal properties of the prepared formulations and neat polymers; Table S2: Summary of the mechanical properties of the prepared formulations and neat polymers (PDF)
