# Supplementary material for: Tailored Plasticization of Bio- and Fossil-Based Polymers Using a Versatile Bioplasticizer Derived from Phenylacetic Acid and Glycerol
Source: ACS Polym Au. 2026 Jan 27;6(1):353–65. doi: 10.1021/acspolymersau.5c00149 (PMC12903500; doi:10.1021/acspolymersau.5c00149)
Supplement: Supplementary file 1 [file lg5c00149_si_001.pdf]

SUPPORTING INFORMATION

Tailored plasticization of bio- and fossil-based polymers using a versatile bioplasticizer derived from phenylacetic acid and glycerol

Laura Martellosio [a,b], Martina Ferri [a,b], Luca Lenzi [a,b], Arianna Tauro [b,c], Andrea Dorigato [b,c], Micaela Degli Esposti [a,b], Davide Morselli [a,b,]* Paola Fabbri [a,b]

[a] *Department of Civil, Chemical, Environmental and Materials Engineering, ALMA MATER STUDIORUM – Università di Bologna, Via Terracini 28, 40131 Bologna, Italy*

[b] *National Interuniversity Consortium of Materials Science and Technology (INSTM), Via Giusti 9, 50121 Firenze, Italy*

[c] *Department of Industrial Engineering, Università di Trento, Via Sommarive 9, 38123 Povo, Italy*

* Corresponding Author:
Davide Morselli
davide.morselli6@unibo.it

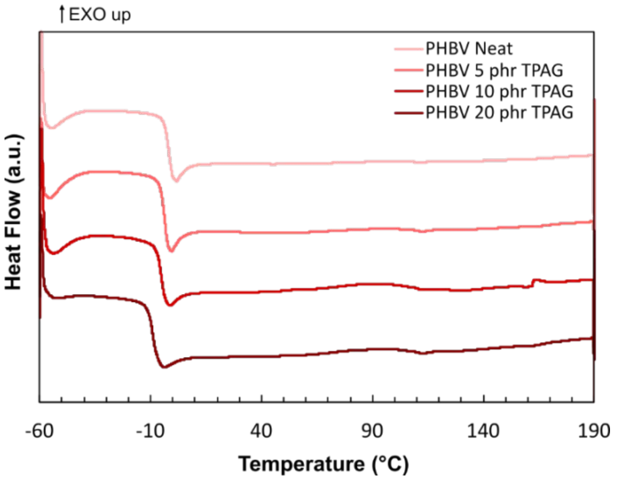

Figure S1. Full scale DSC thermograms recorded from the second heating scan of neat PHBV and related TPAG formulation.

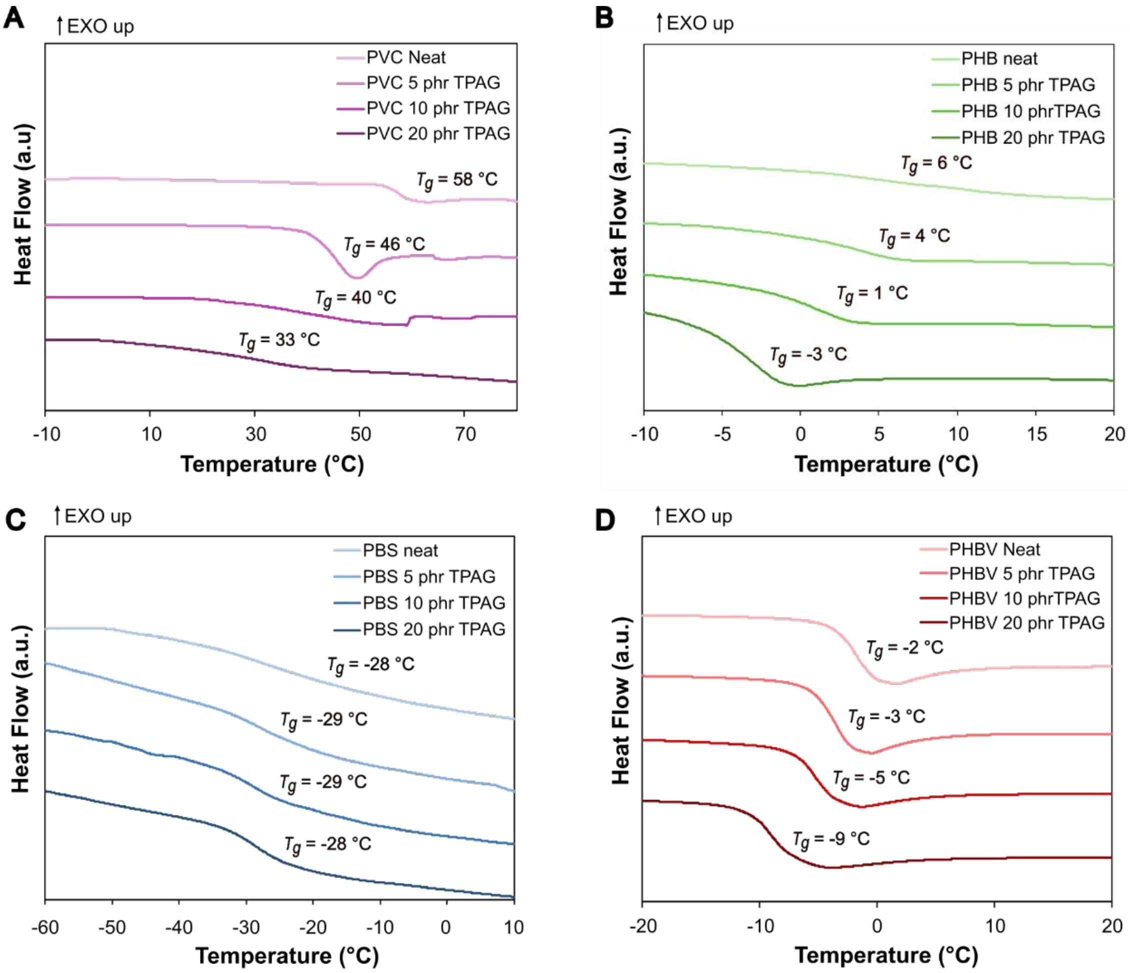

Figure S2. DSC thermograms recorded during the second heating scan at 10°C·min^{-1}, showing glass transition temperatures (T_g) of neat and TPAG formulations of **(A)** PVC, **(B)** PHB, **(C)** PBS and **(D)** PHBV.

Table S1. Glass transition temperature ($T_{g,DSC}$), melting temperature (T_m) and melting enthalpy (ΔH_m) extrapolated from DSC thermograms, crystalline degree (X_c, calculated by Eq. 2), onset temperature (T_{onset}) and maximum degradation temperature (T_{max}) obtained from TGA of neat and plasticized polymers.

**Polymer**	**TPAG content (phr)**	$T_{g,DSC}$ **(°C)**	T_m **(°C)**	ΔH_m **(J·g⁻¹)**	X_c **(%)**	T_{onset} **(°C)**	T_{max} **(°C)**
PVC	0	58	-	-	-	264[1]<br>426[2]	282[1]<br>443[2]
	5	46	-	-	-	-	-
	10	40	-	-	-	-	-
	20	33	-	-	-	260[1]<br>425[2]	290[1]<br>447[2]
PHB	0	6	147<br>172	84	57	250	261
	5	4	147<br>171	77	55	-	
	10	1	147<br>169	75	57	-	
	20	-3	147<br>167	68	58	250	260
PBS	0	-28	115	62	30	356	382
	5	-29	114	67	33	-	-
	10	-29	113	66	35	-	-
	20	-28	112	62	37	351	381
PHBV	0	-2	-	-	-	257	268
	5	-3	-	-	-	-	-
	10	-5	-	-	-	-	-
	20	-9	-	-	-	261	271

[1] First thermal degradation step (dehydrochlorination)

[2] Second thermal degradation step (cracking of the hydrocarbon chains)

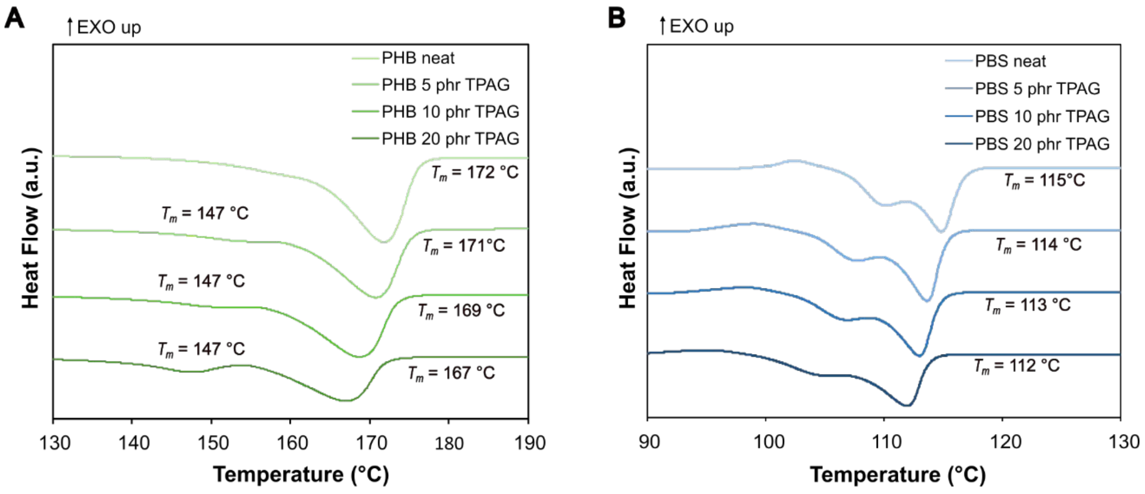

Figure S3. DSC thermograms recorded during the second heating scan at 10°C·min^{-1}, endothermic melting peaks and the relative melting temperatures *(T_m)* of neat and TPAG formulations of (A) PHB and (B) PBS.

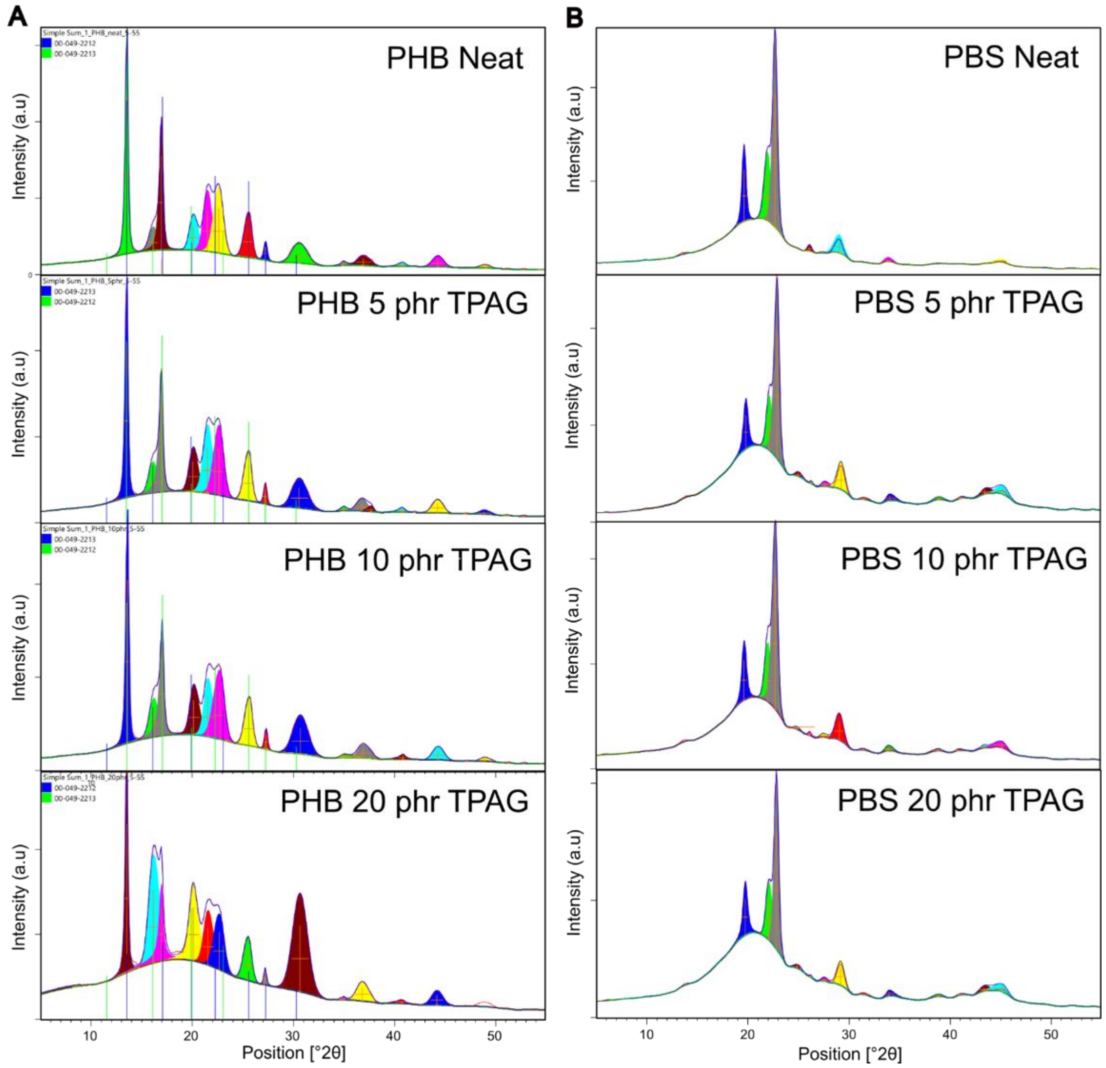

Figure S4. Results of the fitting and deconvolution process obtained from the XRD patterns collected in the 5–55° (2θ) range for neat and TPAG formulations (5, 10 and 20 phr) of **(A)** PHB and **(B)** PBS.

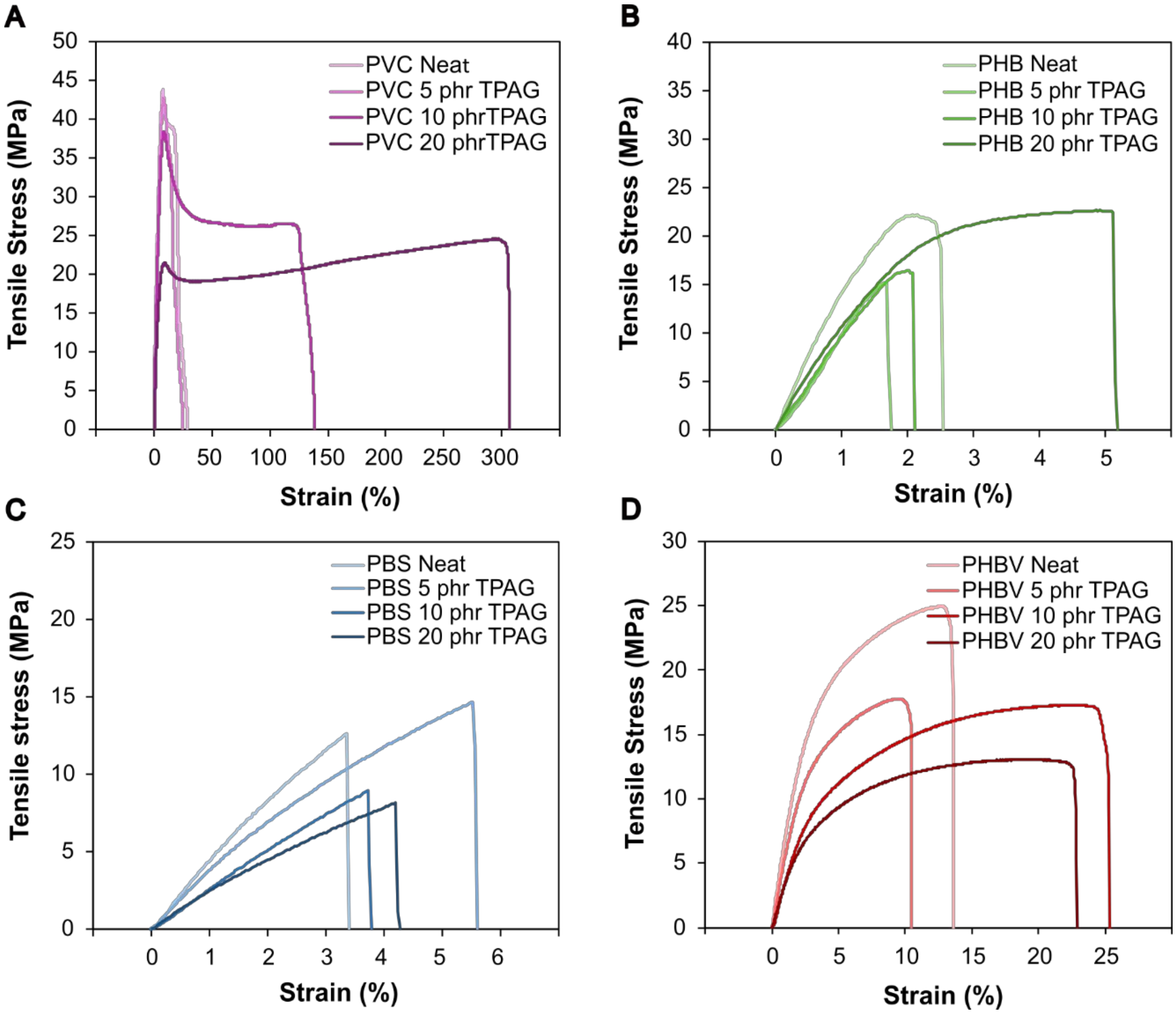

Figure S5. Stress-strain curves of neat and plasticized samples of **(A)** PVC, **(B)** PHB, **(C)** PBS, and **(D)** PHBV.

Table S2. Young's modulus (E) and elongation at break (ε_{break}) obtained from tensile tests of neat and plasticized polymers. E and ε_{break} are expressed as mean value ± standard deviation.

**Polymer**	**TPAG content (phr)**	E **(MPa)**	ε_{break} **(%)**
PVC	0	1346 ± 95	17 ± 4
	5	1036 ± 106	15 ± 3
	10	1107 ± 184	106 ± 61
	20	698 ± 289	349 ± 68
PHB	0	1610 ± 96	2.3 ± 0.2
	5	1009 ± 381	2.1 ± 0.4
	10	1008 ± 329	2.0 ± 0.8
	20	1160 ± 80	5.1 ± 0.9
PBS	0	448 ± 36	2.9 ± 0.8
	5	409 ± 31	5.4 ± 0.2
	10	248 ± 62	3.4 ± 1.5
	20	285 ± 27	4.1 ± 0.4
PHBV	0	736 ± 65	14 ± 1
	5	491 ± 112	10 ± 2
	10	360 ± 108	26 ± 6
	20	311 ± 33	22 ± 1

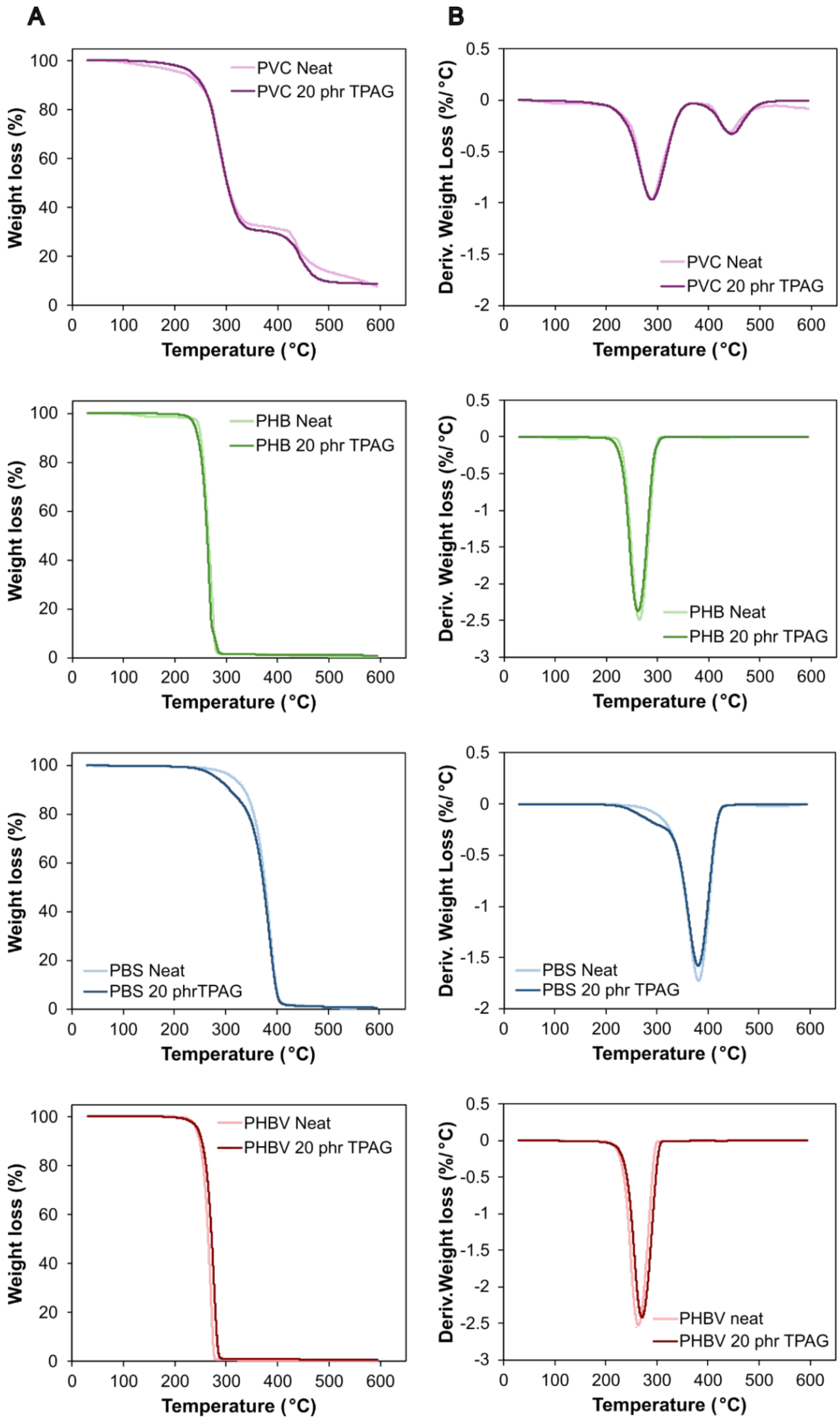

Figure S6. (A) TGA thermograms of PVC, PHB, PBS and PHBV recorded through a temperature ramp of 10 °C·min^{-1} from 30 to 600 °C with a 60 mL·min^{-1} nitrogen flow. **(B)** DTGA curves obtained from TGA thermograms of PVC, PHB, PBS and PHBV.
